# Impact of Body Composition During Neoadjuvant Chemoradiotherapy on Complications, Survival and Tumor Response in Patients With Locally Advanced Rectal Cancer

**DOI:** 10.3389/fnut.2022.796601

**Published:** 2022-01-27

**Authors:** Zhenzhen Liu, Siyi Lu, Yuxia Wang, Xinyi Lin, Peng Ran, Xin Zhou, Wei Fu, Hao Wang

**Affiliations:** ^1^Department of General Surgery, Peking University Third Hospital, Beijing, China; ^2^Department of Radiotherapy, Peking University Third Hospital, Beijing, China

**Keywords:** body composition, rectal cancer, complications, prognosis, tumor response

## Abstract

**Background:**

To explore the impact of body composition before neoadjuvant chemoradiotherapy (pre-NCRT) and after neoadjuvant chemoradiotherapy (post-NCRT) on complications, survival, and tumor response in patients with locally advanced rectal cancer (LARC).

**Methods:**

Patients with LARC who underwent radical surgery after NCRT between Ja 22nuary 2012 and March 2019 were studied. Body composition parameters, including skeletal muscle area (SMA), muscle density (MD), visceral fat area (VFA), total abdominal fat area (TAFA), and subcutaneous fat area (SFA), was identified at the third lumbar vertebra level on computed tomography (CT). The patients were divided into two groups based on the sex-specific quartile values of SMA, MD, VFA, TAFA, SFA, and body composition change. Patient characteristics, short- and long-term postoperative complications, survival, and tumor response were analyzed.

**Results:**

A total of 122 eligible patients were enrolled. Body composition parameters, except MD, were strongly correlated with BMI (*p* < 0.001). Pre-NCRT low MD (*p* = 0.04) and TAFA loss (*p* = 0.02) were significantly correlated with short- and long-term ileus, respectively. Pre-NCRT low SMA was a significant prognostic factor for both disease-free survival (DFS) (HR 2.611, 95% CI 1.129–6.040, *p* = 0.025) and cancer-specific survival (CSS) (HR 3.124, 95% CI 1.030–9.472, *p* = 0.044) in the Cox regression multivariate analysis. Multivariate logistic regression analysis identified post-NCRT SFA (OR 3.425, 95% CI 1.392–8.427, *p* = 0.007) and SFA loss (OR 3.358, 95% CI 1.214–9.289, *p* = 0.02) as independent risk factors for tumor regression grade (TRG) and downstaging, respectively.

**Conclusion:**

Pre-NCRT low MD and TAFA loss were related to a high incidence of short- and long-term ileus, respectively. Pre-NCRT low SMA was a significant prognostic factor for CSS and DFS. Post-NCRT SFA and SFA loss were independent risk factors for TRG and downstaging, respectively.

## Background

Colorectal cancer is one of the most common cancers worldwide and is the second leading cause of cancer-related deaths (1). Rectal cancer accounts for nearly 30% of all colorectal cancers (2). Despite progress in standard treatment for locally advanced rectal cancer (LARC) and neoadjuvant chemoradiotherapy (NCRT) with total mesorectal excision, LARC patients are still burdened by considerable risks of morbidity and metastasis (3–5). Moreover, tumor response after NCRT is a critical reference index for the subsequent treatment and prognosis of patients (6, 7). Hence, preoperative modifiable risk factors that could potentially identify complications, survival prospects, and tumor response in LARC patients are needed to stratify patients with high-risk status and guide tailored treatment.

Cancer-related inflammation and malnutrition are highly prevalent in cancer patients and are essential predictors of complications, survival, and tumor response (8, 9). Patients with cancer-related inflammation and malnutrition are more prone to obtaining a reduced therapeutic effect and experiencing increased chemotherapy toxicity (10–13). Previous studies indicated that a scoring system combining inflammatory and nutritional parameters plays an essential role in predicting outcomes, cancer treatment results and survival (14, 15). Body composition identified from computed tomography (CT) at the third lumbar cross-section of skeletal muscle and fat area is considered an essential biomarker that reflects both inflammatory and nutritional statuses, and its association with cancer outcomes is gaining attention (16, 17). In addition, unlike body mass index (BMI), which neglects the role of sex and is unable to differentiate between muscle mass and fat mass or to characterize the distribution of adipose tissue, body composition could reflect the “real” status of cancer patients more precisely (18–20).

Recently, several meta-analyses have shown the value of CT-based specific profiles of the muscle and adipose parameters (body composition) in predicting short- and long-term outcomes in several cancers (21–23). Skeletal muscle depletion was identified as an independent risk factor for survival in non-metastatic colorectal cancer (13). In rectal cancer, CT-quantified adipose tissue distribution was strongly associated with postoperative complications (24). Furthermore, Chung et al. (25) analyzed 93 LARC patients and found that the change in muscle mass might be a promising parameter to predict overall survival. Notwithstanding, several studies have assessed the relationship between CT-based body composition and LARC, but these studies did not thoroughly assess pre- and post-NCRT body composition and the change in body composition or determine which specific parameters might be risk factors for postoperative morbidity, long-term oncological outcome, and tumor response.

Hence, our study aimed to analyze pre- and post-NCRT body composition parameters and the change in body composition during NCRT to assess the relationship between nutritional status and body composition parameters and to identify whether different body composition parameters could be predictive of short- and long-term complications, survival, and tumor response in a homogenous group of patients with LARC.

## Methods

### Study Population

A total of 122 patients with LARC with prospective follow-up data treated at the Department of General Surgery at Peking University Third Hospital were retrospectively analyzed between January 2012 and March 2018. The inclusion criteria were as follows: (1) pre-NCRT colonoscopy pathology confirming the diagnosis of adenocarcinoma; (2) diagnosis of LARC through pre-NCRT CT and magnetic resonance imaging (MRI); (3) all patients underwent NCRT followed by radical surgery; and (4) complete inpatient data, including pre- and post-NCRT CT scans and follow-up data. The exclusion criteria were as follows: (1) presence of other cancers in addition to rectal adenocarcinoma; (2) presence of lumbar metal implants; and (3) management by a watch and wait strategy after NCRT. Ethical approval was obtained from Peking University Third Hospital (IRB00006761-M2019387), and this study adhered to the tenets of the Declaration of Helsinki. The requirement for informed consent was waived by the Institutional Review Board of Peking University Third Hospital.

### NCRT Treatment

All patients were treated with the same NCRT treatment scheme. The decision to administer NCRT or conduct radical resection was made by a multidisciplinary team, which consisted of surgeons, oncologists, pathologists, and radiologists. Radiation doses ranged from 45 to 50 Gy given across 25 fractions. Radiation was given according to institutional protocols. The oral capecitabine dosage during the whole course of radiotherapy (RT) was 1,650 mg/m^2^ per day. The American Joint Committee on Cancer (AJCC) eighth edition classification standard recommended by the National Comprehensive Cancer Network (NCCN) guidelines was adopted for the pathological staging of the patients. The AJCC tumor regression grade (TRG) definitions were as follows: TRG0, no sign of tumor cells; TRG1, single tumor cell or small groups of tumor cells; TRG2, residual cancer with a desmoplastic response (mild regression); and TRG3, no tumor cells killed. In this study, TRG0-1 was defined as a good response, while TRG2-3 was defined as a poor response. A decline in postoperative staging compared to clinical staging was defined as downstaging.

### Measurement and Definition of Body Composition

We retrospectively measured pre-NCRT (before starting NCRT) and post-NCRT (8–12 weeks after the cessation of NCRT) cross-sectional CT images in the supine position, taken at the level of the third lumbar vertebra (L3). A Java-based open-source image processing software, ImageJ software v1.47i (National Institutes of Health, Bethesda, MD), was used to determine skeletal muscle and fat tissue areas (26). The following tissue Hounsfield unit (HU) thresholds were employed:−29 to 150 HU for skeletal muscle, and −190 to −30 HU for adipose tissue (Supplementary Figure 1) (26). Muscle density (MD) was calculated through the mean HU of the skeletal muscle area (SMA). SMA, visceral fat area (VFA), total abdominal fat area (TAFA), and subcutaneous fat area (SFA) were normalized by the square of height (m^2^). SMA and MD were divided into low and normal groups according to the lowest sex-specific quartile cutoff values, and VFA, TAFA and SFA were divided into high and normal groups according to the highest sex-specific quartile cutoff values (27). The change in body composition was initially expressed as a percentage calculated by (post-NCRT body composition–pre-NCRT body composition)/pre-NCRT body composition × 100. We dichotomized our patients into a body composition loss group and a normal group according to the lowest quartile cutoff values (25).

### Outcome Parameters

Short-term complications included overall complications, ileus, surgical site infection (SII), unplanned reoperation, and Clavien-Dindo (CD) classification of complications (28). Long-term complications included ileus, delayed reversal, reversal failure, radiation proctitis, and anastomotic stricture. Survival outcomes included cancer-specific survival (CSS) and disease-free survival (DFS). CSS was defined as the period from surgical treatment to the date of death caused by rectal cancer. DFS was defined as the period from surgical treatment to tumor recurrence. Tumor response included TRG and tumor downstaging.

### Statistical Analysis

The Kolmogorov–Smirnov method was used to determine the normality of the data. Normally distributed data are expressed as the means ± standard deviations and were analyzed using independent sample *t*-test, while skewed data are expressed as the medians (interquartile ranges) and were analyzed using the Mann-Whitney U test. Categorical variables were analyzed using the chi-square test or Fisher's exact test. Factors that influenced tumor response were assessed using logistic regression, and factors that influenced DFS and CSS were assessed using Cox regression. Potential risk factors (*p* < 0.1) were adopted for the multivariate analysis with the backward stepwise method, following the results of the univariate analysis. Survival curves were drawn using the Kaplan–Meier method owing to the significant difference observed in the follow-up time of the patients; thus, all survival analyses were targeted at the cumulative survival rate of the patients. Time-dependent receiver operating characteristic (ROC) analysis to compare the prognostic values of the markers for DFS and CSS was performed by the “timeROC” package in R version 3.5.2. All statistical analyses were conducted using SPSS Statistics 24.0 (IBM Corporation, Armonk, NY, USA). A *p*-value of < 0.05 was recognized as statistically significant.

## Results

### Patient Characteristics

**Figure 1:**
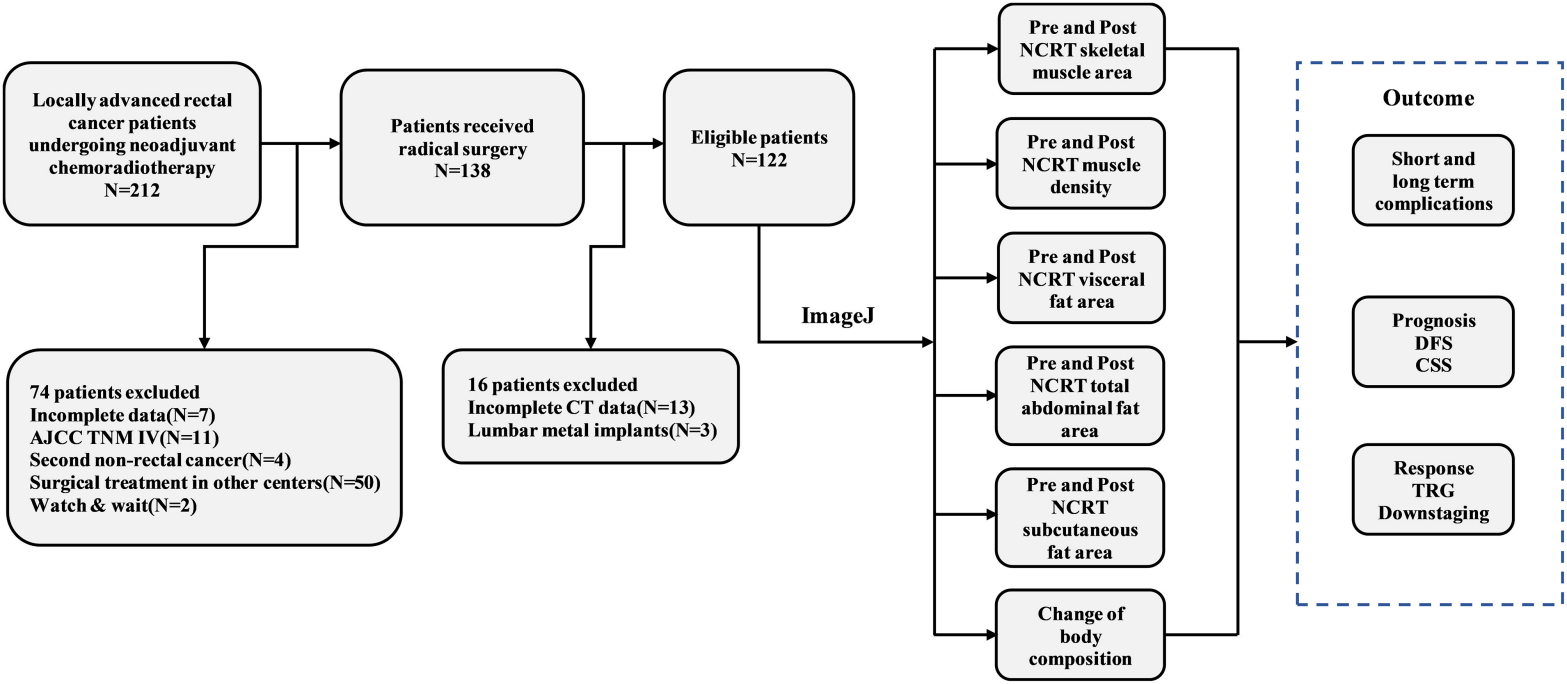
Flow chart of the study.

According to the inclusion and exclusion criteria, 122 patients were eventually enrolled in the study. A detailed flow chart of the patient selection process and outcomes is shown in Figure 1. Among the study population, 88 patients were male (71.5%), with a mean age of 60 years (range 22–82). The mean BMI was 23.9 kg/m^2^ (range 15.2–32.9) for men and 24.4 kg/m^2^ (range 19.1–30.1) for women. Sixty-three (43.7%) patients had tumor size > 4 cm, while 75 (54.3%) had tumor size ≤ 4 cm. Thirty-nine (32.0%) patients had tumors in the lower rectum, while the remaining 83 (68.0%) patients had tumors in the mid-high rectum. A total of 24 (19.7%) patients had clinical stage T4 disease, and 91 (74.6%) patients had clinically positive lymph nodes. Eighteen (14.8%) patients achieved ypT0N0M0 after NCRT, and 89 (76.6%) patients achieved downstaging after NCRT. According to the four-tier AJCC-TRG system, 72 (59%) patients were TRG0-1, while 50 (41%) patients were TRG2-3. The detailed baseline clinicopathological characteristics of the patients are shown in Supplementary Table 1.

### Impact of Neoadjuvant Therapy on Body Composition

**Figure 2:**
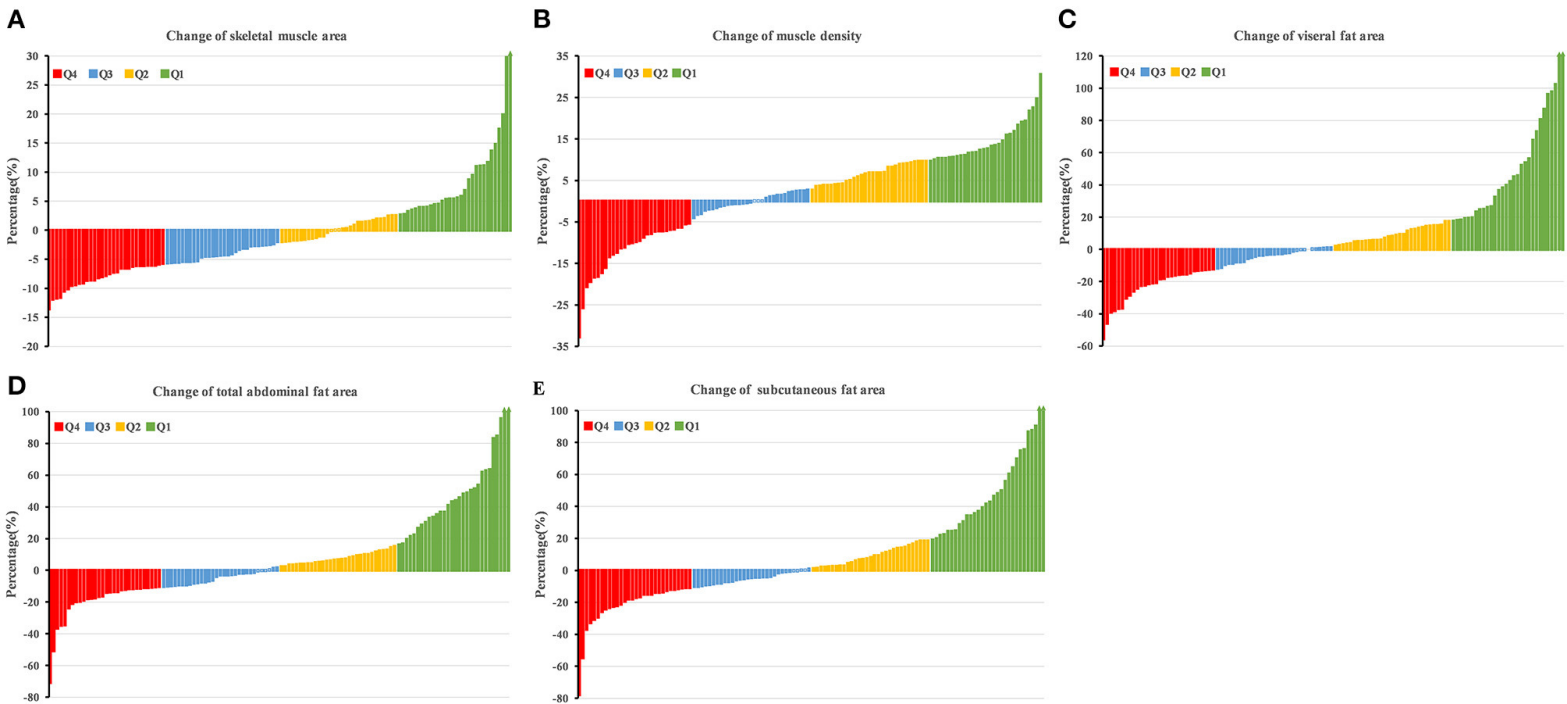
Change of skeletal muscle area **(A)**, muscle density **(B)**, visceral fat area **(C)**, total abdominal fat area **(D)**, subcutaneous fat area **(E)** during NCRT. Q1, Q2, Q3, and Q4 have represented the highest quartile, 50–75%, 25–50%, and lowest quartile, respectively.

The median pre-NCRT SMA, MD, VFA, TAFA, and SFA were 46.47 cm^2^/m^2^, 37.04 HU, 48.99 cm^2^/m^2^, 103.12 cm^2^/m^2^, and 43.46 cm^2^/m^2^, respectively, while the median of post-NCRT SMA, MD, VFA, TAFA, and SFA were 45.88 cm^2^/m^2^, 37.75 HU, 46.93 cm^2^/m^2^, 104.20 cm^2^/m^2^, and 45.35 cm^2^/m^2^. No statistically significant difference was observed between pre-NCRT and post-NCRT body composition (*p* > 0.05). The median changes in SMA, MD, VFA, TAFA, and SFA were −0.65, 2.29, 9.4, 8.24, and 9.67%, respectively. Overall, the distribution of % change in body composition during NCRT is shown in Figure 2. The detailed body composition parameters and the change in body composition of LARC patients are shown in Supplementary Table 2.

### Body Composition and Nutritional Status (BMI, ALB, FIB, and HB)

**Figure 3:**
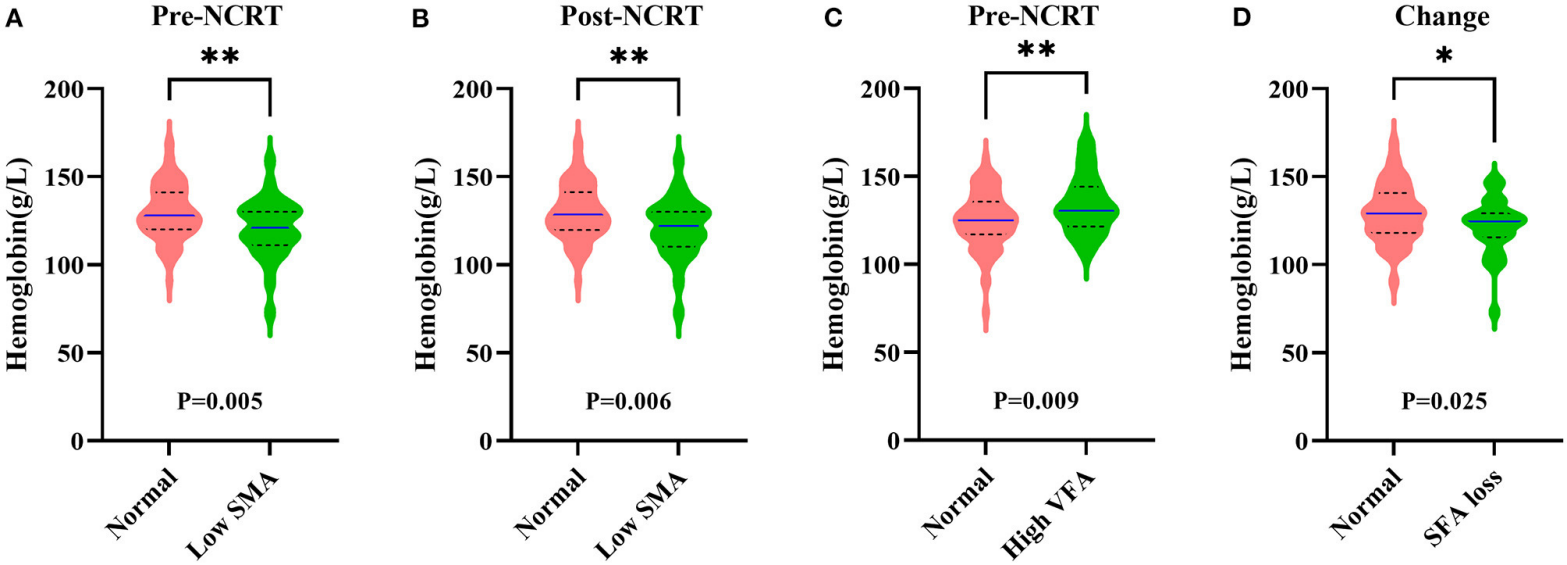
Correlation among hemoglobin and pre-NCRT skeletal muscle area **(SMA) (A)**, post-NCRT SMA **(B)**, pre-NCRT visceral fat area **(VFA) (C)**, subcutaneous fat area **(SFA)** loss **(D)**. The *symbol indicates *p* < 0.05 and **symbol indicates *p* < 0.01.

We further explored the relationship between body composition and nutrition status. Except for pre- and post-NCRT MD, BMI was strongly correlated with pre- and post-NCRT body composition (*p* < 0.001; Supplementary Table 3) and weakly correlated with the change in body composition (*p* > 0.05). There was no significant difference in albumin (ALB) for body composition and change in body composition. Fibrinogen (FIB) was only associated with pre-NCRT SMA (*p* = 0.041). With regard to hemoglobin (HB), there were significant differences in the pre- and post-NCRT low SMA groups (*p* = 0.005; *p* = 0.006), pre-NCRT high VFA group (*p* = 0.009), SFA loss group (*p* = 0.025) and normal group according to the Mann-Whitney U test (Figure 3).

### Short- and Long-Term Complications and Body Composition

A chi-square test was conducted to determine whether body composition was closely correlated with short- and long-term complications. All short- and long-term complication outcomes are included in Table 1. Twenty-six (21.3%) patients experienced a short-term complication, and the rates of ileus, SSI, unplanned reoperation, and CD>III were 9% (11 cases), 9.8% (12 cases), 4.1% (5 cases), and 8.2% (10 cases), respectively. Among all body composition parameters, pre-NCRT low MD (p = 0.04) was related to short-term ileus. The other indicators were not associated with short-term complications. Concerning long-term complications, 10 (8.2%) of 122 patients experienced long-term ileus, while 7 (10.3%) of 68 patients who underwent Dixon operation suffered from radiation proctitis and anastomotic stricture. Of the 63 patients who underwent preventive diverting stoma, 8 (12.7%) failed to undergo reversal. A total of 54 patients underwent stoma reversal, and 37 (68.5%) patients' reversal later than 6 months after surgery was considered delayed. Only TAFA loss (*p* = 0.02) was associated with long-term ileus.

**Table 1:** Correlation of body composition and short- and long-term complications.

**Variable**	**Short-term complications**					**Long-term complications**				
	**Complications *N* = 26**	**Ileus *N* = 11**	**SSI *N* = 12**	**Unplanned reoperation *N* = 5**	**CD > III *N* = 10**	**Ileus *N* = 10**	**Reversal delayed *N* = 54**	**Reversal failure *N* = 8**	**Radiation proctitis *N* = 7**	**Anastomotic stricture *N* = 7**
	***P*** **-value** ^**a**^	***P*** **-value** ^**b**^	***P*** **-value** ^**b**^	***P*** **-value** ^**b**^	***P*** **-value** ^**b**^	***P*** **-value** ^**b**^	***P*** **-value** ^**a**^	***P*** **-value** ^**b**^	***P*** **-value** ^**b**^	***P*** **-value** ^**b**^
**Pre-NCRT**										
Low SMA	0.758	1.000	1.000	0.419	0.430	1.000	0.537	1.000	0.607	1.000
Low MD	0.755	**0.040**	0.750	1.000	0.975	0.425	0.409	0.781	1.000	1.000
High VFA	0.474	1.000	0.750	0.178	0.975	0.462	0.368	0.315	0.386	1.000
High TAFA	0.840	0.376	0.698	0.774	0.975	0.462	0.368	0.315	0.344	1.000
High SFA	0.755	0.880	1.000	1.000	0.975	0.975	1.000	1.000	0.949	0.949
**Post-NCRT**										
Low SMA	0.360	1.000	1.000	1.000	0.926	0.511	0.465	1.000	0.800	1.000
Low MD	0.928	0.659	0.808	1.000	1.000	0.511	1.000	0.618	0.371	1.000
High VFA	0.840	1.000	1.000	1.000	1.000	0.462	0.625	1.000	0.307	0.949
High TAFA	0.840	1.000	1.000	0.439	0.462	0.462	1.000	0.963	0.307	0.949
High SFA	0.064	0.559	0.698	1.000	0.975	0.462	1.000	1.000	0.386	1.000
**Change**										
SMA loss	0.680	0.659	0.255	0.845	1.000	0.159	0.683	1.000	1.000	0.720
MD loss	0.409	1.000	0.274	0.774	0.975	0.462	0.138	1.000	1.000	1.000
VFA loss	0.474	0.880	0.750	0.178	0.425	0.118	0.845	1.000	0.949	1.000
TAFA loss	0.474	1.000	0.750	0.178	0.425	**0.020**	1.000	0.700	0.872	0.872
SFA loss	0.219	1.000	0.306	1.000	1.000	1.000	0.611	1.000	1.000	1.000

*SSI, surgical site infection; CD, Clavien-Dindo classification; SMA, skeletal muscle area; MD, muscle density; VFA, visceral fat area; TAFA, total abdominal fat area; SFA, subcutaneous fat area*.

a *Chi-square test*.

b *Fisher's exact test*.

### Time-Dependent ROC Curve of Body Composition and Change in Body Composition

Time-dependent ROC analysis was conducted to compare the ability of body composition to predict DFS and CSS. In the first, third, fourth, fifth, and sixth years after surgery, the AUCs of pre-NCRT SMA for predicting DFS continued to be superior to those of other parameters (Supplementary Figure 2A). Meanwhile, the time-dependent ROC curve for CSS showed that pre-NCRT SMA has a relatively stable ability in predicting CSS (Supplementary Figure 2B). The AUCs of pre-NCRT SMA for predicting 1-, 2-, 3-, 4-, 5-, and 6-year DFS were 0.678, 0.549, 0.544, 0.621, 0.64, and 0.626, respectively. Meanwhile, the AUCs of pre-NCRT SMA for predicting 2-, 3-, 4-, 5-, 6- and 7-year CSS were 0.537, 0.593, 0.649, 0.608, 0.15, and 0.744, respectively.

### Long-Term Outcomes and Body Composition

**Figure 4:**
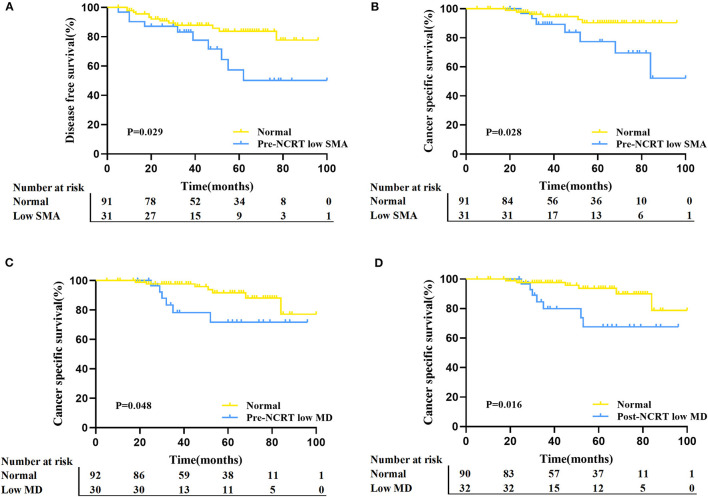
Comparison of DFS and CSS between the different status of body composition in LARC patients. **(A)** Kaplan–Meier analysis for DFS rate between normal and pre-NCRT low SMA groups in LARC patients (*p* = 0.029). **(B)** Kaplan–Meier analysis for the CSS rate between normal and pre-NCRT low SMA groups in LARC patients (*p* = 0.028). **(C)** Kaplan–Meier analysis for CSS rate between normal and pre-NCRT low MD groups in LARC patients (*p* = 0.048). **(D)** Kaplan–Meier analysis for the CSS rate between normal and post-NCRT low MD in LARC patients (*p* = 0.016).

The follow-up time ranged from 5 to 100 months, and the median follow-up time was 46.5 months. Thirteen (10.7%) patients had died at the last follow-up, and local recurrence with or without metastasis occurred in 23 (18.9%) patients among the 122 enrolled patients. With regard to DFS, pre-NCRT low SMA (*p* = 0.029) was significantly correlated with poor DFS according to Kaplan-Meier analysis (Figure 4A), and the cumulative 5-year DFS rate of pre-NCRT low SMA was 57.3%. Regarding CSS, pre-NCRT SMA and pre- and post-NCRT MD could distinguish patients with poor CSS (Figures 4B–D), and the cumulative 5-year DFS rates were 77.3, 71.7, and 67.6%, respectively. The other body composition parameters failed to differentiate survival in LARC patients (Supplementary Figures 3, 4).

Cox regression analysis was conducted further to demonstrate the prognostic value of body composition. Univariate analysis showed that clinical T stage, clinical lymph node status, and pre-NCRT SMA were significantly associated with DFS (Table 2). Multivariate analysis indicated that both pre-NCRT low SMA (HR 2.611, 95% CI 1.129–6.040, *p* = 0.025) and clinical stage T4 (HR 2.811, 95% CI 1.165–6.780, *p* = 0.021) were independent prognostic factors of poor DFS in LARC patients undergoing radical surgery following NCRT. Meanwhile, univariate analysis showed that pre-NCRT SMA and post-NCRT MD were also significantly associated with CSS (Table 2). Subsequent multivariate analysis showed that pre-NCRT low SMA (HR 3.124, 95% CI 1.030–9.472, *p* = 0.044) and post-NCRT low MD (HR 3.532, 95% CI 1.181–10.557, *p* = 0.024) were independent risk factors for CSS (Table 2).

**Table 2:** Cox proportion independent predictors of DFS and CCS in LARC patients.

	**DFS**	**CSS**						
	**Univariate**	**Multivariate**	**Univariate**	**Multivariate**				
	**HR (95%CI)**	***P-*** **value**	**HR (95%CI)**	***P-*** **value**	**HR (95%CI)**	***P*** **-value**	**HR (95%CI)**	***P*** **-value**
Gender (male vs. female)	0.600 (0.222–1.623)	0.314	-	-	0.324 (0.071–1.486)	0.147	-	-
Age, years	1.008 (0.976–1.042)	0.617	-	-	1.022 (0.976–1.070)	0.358	-	-
BMI (kg/m^2^)	0.945 (0.831–1.074)	0.387	-	-	0.884 (0.774–1.052)	0.165	-	-
Tumor size (>4 vs. ≤4 cm)	2.915 (0.865–9.826)	0.084	-	-	4.844 (0.628–37.383)	0.130	-	-
Surgery procedure	-	0.592	-	-	-	0.270	-	-
Miles vs. hartmann	0.574 (0.168–1.962)		-	-	0.588 (0.140–2.474)	0.469	-	-
Dixon vs. hartmann	0.567 (0.183–1.758)		-	-	0.310 (0.073–1.323)	0.114	-	-
Tumor location			-	-	-	-	-	-
Low vs. mid-high	0.774 (0.305–1.965)	0.589	-	-	0.737 (0.203–2.681)	0.643	-	-
cT (cT4 vs. cT2-3)	3.066 (1.291–7.283)	**0.011**	2.811 (1.165–6.780)	**0.021**	2.966 (0.956–9.197)	0.060	2.944 (0.940–9.226)	0.064
cN (negative vs. positive)	4.539 (1.062–19.400)	**0.041**	3.820 (0.888–16.437)	0.072	34.598 (0.217–5513.741)	0.171	-	-
ypTNM (0 vs. I–III)	0.457 (0.107–1.956)	0.291	-	-	0.431 (0.56–3.349)	0.421	-	-
CEA (>5 vs. ≤5 ng/L)	2.404 (0.947–6.105)	0.065	-	-	2.488 (0.755–8.204)	0.134	-	-
Pre-NCRT low SMA vs. normal	2.429 (1.063–5.549)	**0.035**	2.611 (1.129–6.040)	**0.025**	3.200 (1.072–9.558)	**0.037**	3.124 (1.030–9.472)	**0.044**
Pre-NCRT low MD vs. normal	2.070 (0.895–4.789)	0.089	-	-	2.880 (0.963–8.619)	0.059	-	-
Post-NCRT low MD vs. normal	-	-	-	-	3.532 (1.181–10.557)	**0.024**	3.006 (1.003–9.008)	**0.049**

*HR, hazard ratio; CI, cofidence interval; cT, clinical T stage; cN, clinical N status; ypTNM, post neoadjuvant pathological TNM stage; CEA, carcinoembryonic antigen; SMA, skeletal muscle area; MD, muscle density. The bold values represent P < 0.05*.

### Tumor Response and Body Composition

**Figure 5:**
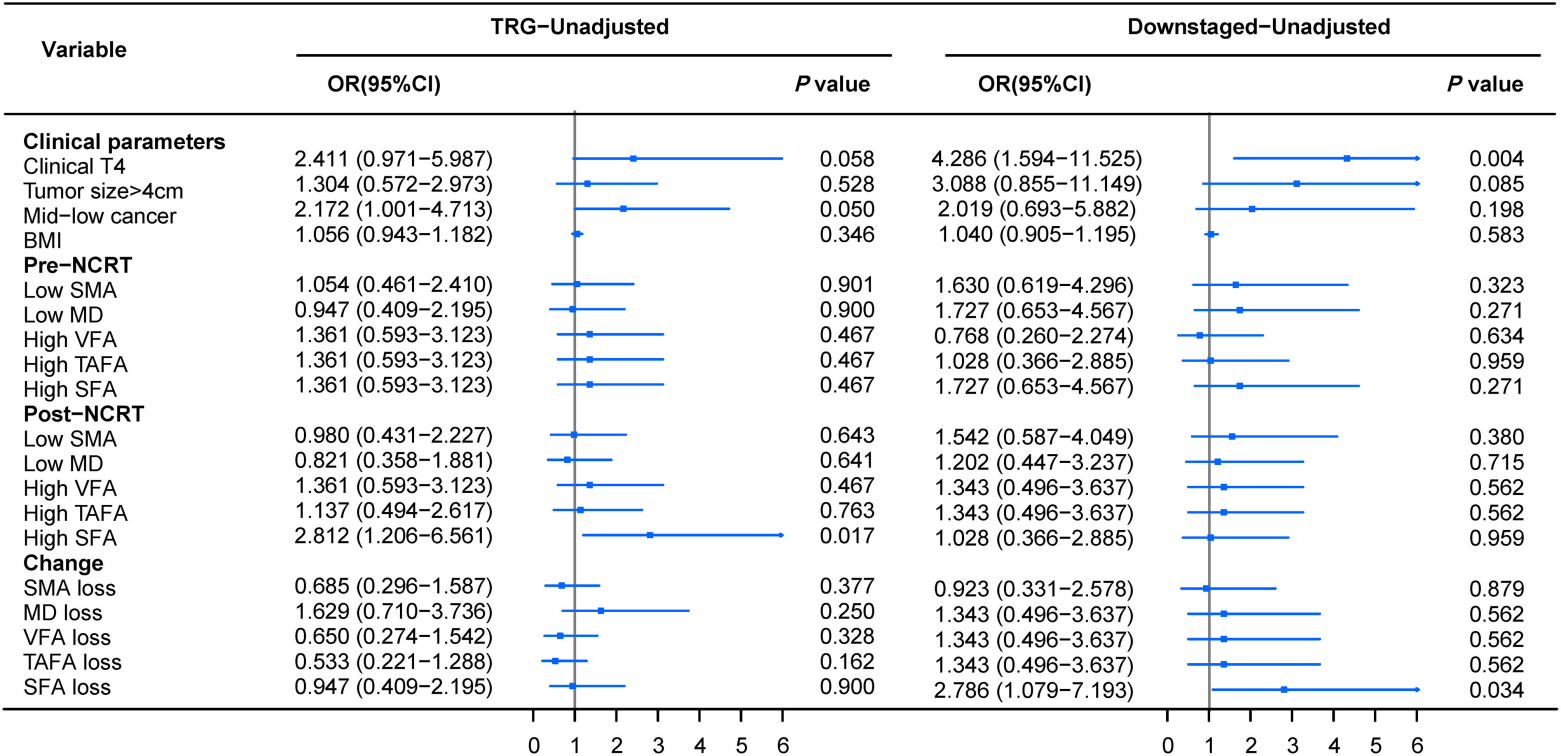
Forrest plot of unadjusted logistic regression to assess the discrimination ability of body composition for tumor TRG and downstaging.

Finally, logistic regression analysis was performed based on TRG and downstaging to further determine the clinical utility of body composition in predicting tumor response to NCRT. In the univariate logistic regression analysis of TRG, post-NCRT high SFA was associated with a poor response, while the other body composition parameters were not (Figure 5). Concerning downstaging, cT4 and SFA loss were strongly correlated with poor downstaging (Figure 5). In multivariate logistic regression analysis, post-NCRT low SFA (OR 3.425, 95% CI 1.392–8.427, *p* = 0.007) and SFA loss (OR 3.358, 95% CI 1.214–9.289, *p* = 0.02) remained significantly associated with TRG and downstaging, respectively. Detailed data are shown in Tables 3, 4.

**Table 3:** Multivariate logistic regression analysis for TRG in LARC patients.

**Variables**	**Score**	***N***		
			**Multivariate**	
			**OR (95%CI)**	***P*** **-value**
cT	cT2-3	98	1 (-)	-
	cT4	24	3.801 (1.413–10.224)	**0.008**
Tumor location	Mid-High	83	1 (-)	-
	Low	39	2.666 (1.153–6.163)	**0.022**
Post-High SFA	Low	92	1 (-)	-
	High	30	3.425 (1.392–8.427)	**0.007**

*OR, odds ratio; CI, confidence interval; cT, clinical T stage; SFA, subcutaneous fat area. The bold values represent P < 0.05*.

**Table 4:** Multivariate logistic regression analysis for downstaged LARC patients.

**Variables**	**Score**	***N***		
			**Multivariate**	
			**OR (95%CI)**	***P*** **-value**
cT	cT2-3	98	1 (-)	-
	cT4	24	5.003 (1.765–14.188)	**0.002**
Tumor size	≤4 cm	33	1 (-)	-
	>4 cm	89	0.205 (0.112–1.600)	0.205
SFA change	Normal	92	1 (-)	-
	Loss	30	3.358 (1.214–9.289)	**0.020**

*OR, odds ratio; CI, confidence interval; cT, clinical T stage; SFA, subcutaneous fat area. The bold values represent P < 0.05*.

## Discussion

We used CT-based pre- and post-NCRT body composition and change in body composition to explore potential markers to predict short- and long-term complications, survival, and tumor response. First, no significant change was observed in body composition during NCRT. Second, we found a strong correlation between nutritional status and specific body composition parameters. Third, we found that pre-NCRT MD and TAFA loss significantly correlated with short- and long-term ileus, respectively. Fourth, we found that pre-NCRT low SMA was an independent risk factor for both DFS and CSS through Cox regression analysis. Finally, through logistic regression, we found that subcutaneous fat tissue and its change during NCRT were independent risk factors for TRG and downstaging, respectively. This study demonstrated that specific indicators of body composition are promising predictors of specific types of complications, survival, and tumor response in LARC patients.

In previous studies, BMI was widely adopted to predict the postoperative short- and long-term outcomes of cancer patients because it is relatively easy to collect in large studies; however, it is also well known to be a less effective measure of body composition, overlooking the role of sex and the proportions of muscle and fat tissue (18–20). Our findings also reflect the same phenomenon as previous studies, as BMI showed weak correlations with survival and tumor response. Conversely, in our study, body composition showed a good ability to predict postoperative complications, survival, and tumor response in LARC patients. Additionally, abdominal CT examinations are routinely performed pre- and post-NCRT, confirming that body composition is a better standard parameter for LARC patients. CT-based body composition analyses have been performed in the clinic in the European population for decades, and a common cutoff value for body composition is well defined. However, the body composition of the Asian population is significantly different from that of the European population. The optimal cutoff value for body composition in the Asian population is still unclear. Miyamoto et al. found that the sex-specific quartile cutoff value of body composition was suitable for the Asian population, and skeletal muscle depletion according to this cutoff value was closely correlated with high mortality in colorectal cancer (27). For practical reasons to improve discrimination, we dichotomized our patients into different groups according to the sex-specific quartile value.

Sheikhbahaei et al. reported that prostate cancer patients suffer from a significant reduction in muscle mass and an increase in subcutaneous adiposity during NCRT (29). Interestingly, no apparent change in body composition was observed in our study, which is consistent with the findings of Chung et al.'s and De Nardi et al.'s study in LARC patients (25, 30). This is probably due to the difference in the timing of post-NCRT imaging. In Chung et al.'s, De Nardi et al.'s and our study, all patients underwent post-NCRT imaging 4–12 weeks after NCRT compared with 3–12 months in Sheikhbahaei et al.'s study. This finding indicates no significant difference in body composition in the population receiving neoadjuvant therapy in a short period.

Recently, a study of 1,630 stage I to III colon cancer patients indicated that low SMA and low MD were associated with a longer length of stay and a higher risk of postsurgical complications (31). A published study by Heus et al. that measured visceral obesity at L3-L4 of the preoperative CT scan demonstrated that VFA ≥100 cm^2^ was associated with a higher occurrence of complications in patients with advanced ovarian cancer undergoing cytoreductive surgery (32). These studies all suggested that body composition parameters might be promising predictors of postsurgical complications in cancer patients. However, these findings were restricted to complications within 30 days after surgery, and the correlation between long-term postoperative morbidity and body composition remains unclear. Hence, we comprehensively analyzed the relationship between body composition and short- and long-term complications. Pre-NCRT low MD was correlated with a higher incidence of short-term ileus in LARC patients, while TAFA loss was correlated with a higher incidence of long-term ileus. However, we did not find an association between muscle mass and short- and long-term complications. In line with our results, Chung et al. and De Nardi et al. also showed no association between skeletal muscle and postoperative complications, and explained that due to the shorter gap between CT scans and surgery (25, 30). The change in muscle mass was not been observed in that short gap, thus significant impact on muscle mass in complications could not be observed.

To explore the relationship between body composition and prognosis in LARC, we conducted a multivariate analysis of DFS and CSS. Pre-NCRT low SMA was an independent risk factor for both DFS (HR 2.611, 95% CI 1.129–6.040, *p* = 0.025) and CSS (HR 3.124, 95% CI 1.030–9.472, *p* = 0.044). Patients with pre-NCRT low SMA had a significantly lower DFS and CSS than normal patients, which was consistent with the findings of previous studies on body composition (25, 33, 34). However, other adipose-based indicators did not show the same phenomenon in our study, which indicated that obesity might cause some difficulty in surgery and lead to a higher complication rate (35), but obesity does not cause a decline in survival. For patients with muscle depletion, it may be challenging to tolerate the whole process of radiotherapy and chemotherapy, resulting in a decrease in the treatment intensity of patients (10–13). Furthermore, in our study, patients with pre-NCRT low SMA were strongly correlated with low HB levels and high FIB levels, indicating that pre-NCRT low SMA is closely associated with malnutrition and inflammation in LARC patients. Cancer-related inflammation and malnutrition are highly prevalent in cancer patients and serve as vital survival predictors (8, 9). In addition, skeletal muscle depletion underlines insulin resistance and chronic inflammation in breast cancer, leading to cancer progression and poor survival (36). The above situation may be the reason why pre-NCRT low SMA was associated with unfavorable survival in our study.

To our knowledge, tumor response plays an essential role in treating LARC patients (6), but there is still a lack of research on the relationship between body composition and tumor response in LARC. Recently, some researchers have started to focus on this issue. Lin et al. established a novel model using pre-NCRT MD and SMA loss that was proposed to predict the tumor response in locally advanced gastric cancer with an area under the curve of 0.764 (37). Omarini et al. reported that visceral adiposity was closely involved in chemosensitivity in breast cancer, and high VFA was a negative predictive factor for pathological complete response (38). However, De Nardi et al. reported that both SMA, SFA and VFA variation after NCRT did not correlated to TRG in LARC (30). The lack of significative in this study might be caused by the small sample size, only 52 patients were included. Our results suggest that post-NCRT SFA (OR 3.425, 95% CI 1.392–8.427, *p* = 0.007) was an independent risk factor for TRG, while SFA loss (OR 3.358, 95% CI 1.214–9.289, *p* = 0.02) was an independent risk factor for downstaging. The unfavorable impact of SFA on TRG might be attributed to the following reasons. Fat tissue, previously thought to only store and mobilize lipids, is now gradually being recognized as a complex secretory organ that can produce cytokines (interleukin-1, interleukin-6, and tumor necrosis factor-α) (39), cause a systemic inflammatory response and regulate FIB levels to cause NCRT resistance (40). SFA loss reflects a rare condition called lipodystrophy, which is associated with secondary metabolic resistance syndrome, including hyperlipidemia and insulin resistance, and patients with lipodystrophy are more prone to a reduced therapy effect (41). This indicates that significant SFA loss may be a mechanism underlying poor downstaging in patients with LARC who underwent NCRT.

Some limitations exist in this study. First, this study was a single-center retrospective study, so some selection bias inevitably exists. Second, due to this study's relatively small sample size, some research endpoints only showed a tendency related to body composition but did not show a significant difference. More patients should be included in the future, and the follow-up time should be extended to verify these findings. Third, this study explored body composition at only two time points, pre-NCRT and post-NCRT, without considering the postoperative time point. Body composition changes over time. It would be necessary to determine which specific time point may accurately reflect the outcome of patients. Finally, we chose sex-specific quartiles as a cutoff value according to a previous study. Further studies may be needed to confirm our results to clarify that this cutoff value is suitable for the Asian population.

In summary, this study is the first to comprehensively analyze pre- and post-NCRT body composition parameters and the change in body composition during NCRT and to assess their relationships with short- and long-term complications, survival, and tumor response in a homogenous group of patients with LARC. A better understanding of CT-based body composition may be key to optimizing patient conditions and allowing more accurate preoperative risk stratification.

## Conclusion

In conclusion, CT-based body composition parameters could predict short- and long-term complications, long-term survival, and tumor response in LARC. Of importance, pre-NCRT SMA status has significant prognostic value for individuals with LARC.

## Data Availability Statement

The raw data supporting the conclusions of this article will be made available by the authors, without undue reservation.

## Ethics Statement

Ethical approval was obtained from Peking University Third Hospital (IRB00006761-M2019387), and this study adhered to the tenets of the Declaration of Helsinki. Written informed consent for participation was not required for this study in accordance with the national legislation and the institutional requirements. Written informed consent was not obtained from the individual(s) for the publication of any potentially identifiable images or data included in this article.

## Author Contributions

ZL, SL, and YW collected and analyzed data and wrote the manuscript. XL and PR contributed to data collection. YW and HW contributed to follow-up. XZ and HW provided intellectual contributions. HW, XZ, and WF supervised the project, discussed data analysis, and reviewed the manuscript.

## Funding

This work was supported by grants from the National Natural Science Foundation of China (Nos. 91959110 and 82003153), Peking University Medicine Seed Fund for Interdisciplinary Research, combining radiotherapy, and a novel nano-system cGAMP-nanodiamond for immunotherapy (No. BMU2021MX027).

## Conflict of Interest

The authors declare that the research was conducted in the absence of any commercial or financial relationships that could be construed as a potential conflict of interest.

## Publisher's Note

All claims expressed in this article are solely those of the authors and do not necessarily represent those of their affiliated organizations, or those of the publisher, the editors and the reviewers. Any product that may be evaluated in this article, or claim that may be made by its manufacturer, is not guaranteed or endorsed by the publisher.

## Supplementary Material

Supplementary Figure 1 CT-based cross-sectional image of the third lumbar (L3) measured for SMA **(A)**, VFA **(B)**, TATA **(C)**, and SFA **(D)** using ImageJ software v1.47i (National Institutes of Health, Bethesda, MD). The area delineated by the yellow solid line represents the region of interest.

Supplementary Figure 2 Time-dependent ROC of body composition and the change of body composition for DFS **(A)** and CSS **(B)**.

Supplementary Figure 3 Kaplan-Meier analysis for DFS according to body composition and change of body composition.

Supplementary Figure 4 Kaplan-Meier analysis for CSS according to body composition and change of body composition.
