# Supplementary figures and images for: Impact of Body Composition During Neoadjuvant Chemoradiotherapy on Complications, Survival and Tumor Response in Patients With Locally Advanced Rectal Cancer
Source: Front Nutr. 2022 Jan 27;9:796601. doi: 10.3389/fnut.2022.796601 (PMC8830534; doi:10.3389/fnut.2022.796601)

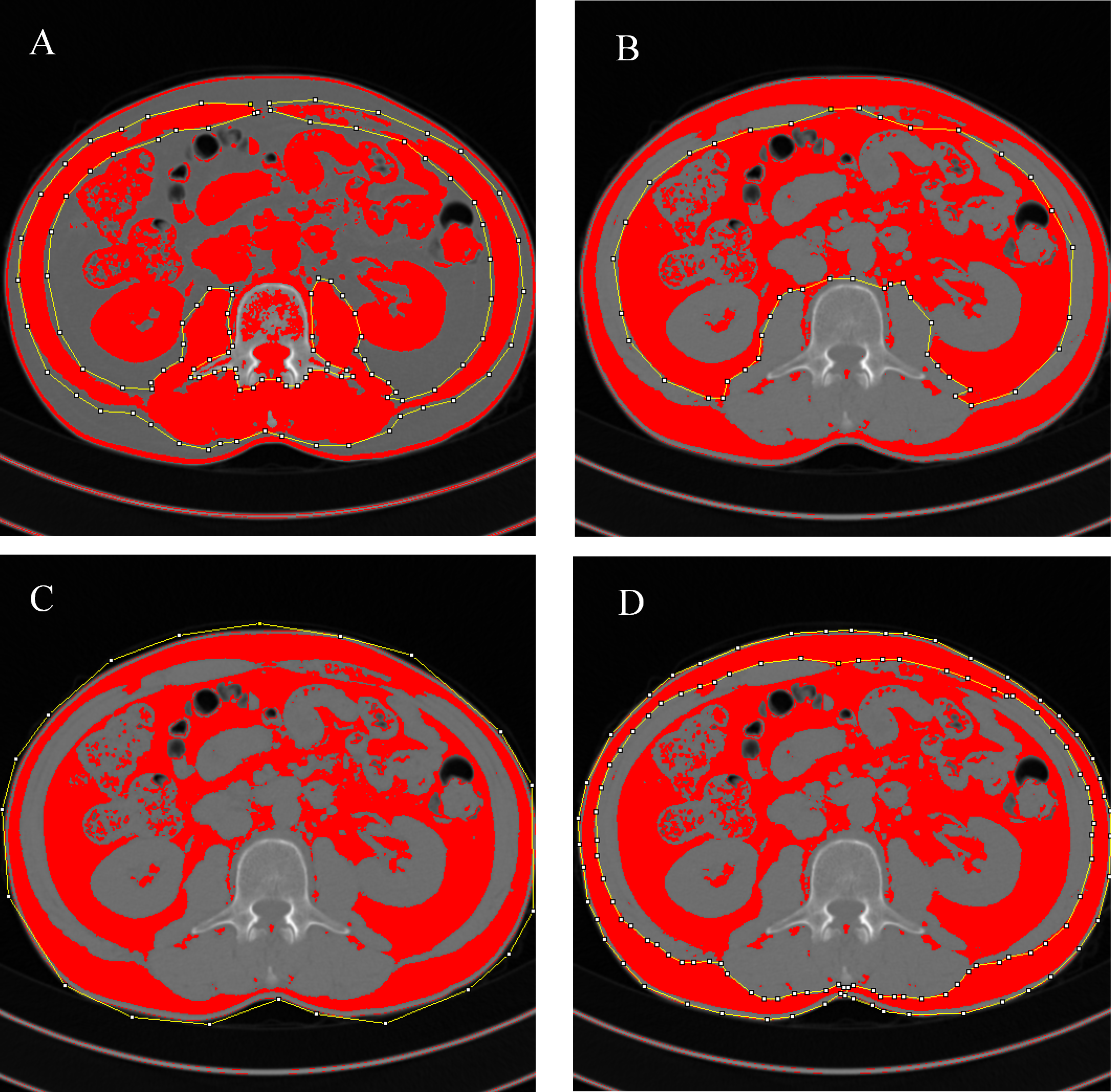

Supplement: Supplementary Figure 1 — CT-based cross-sectional image of the third lumbar (L3) measured for SMA (A), VFA (B), TATA (C), and SFA (D) using ImageJ software v1.47i (National Institutes of Health, Bethesda, MD). The area delineated by the yellow solid line represents the region of interest. [file Image_1.TIF]

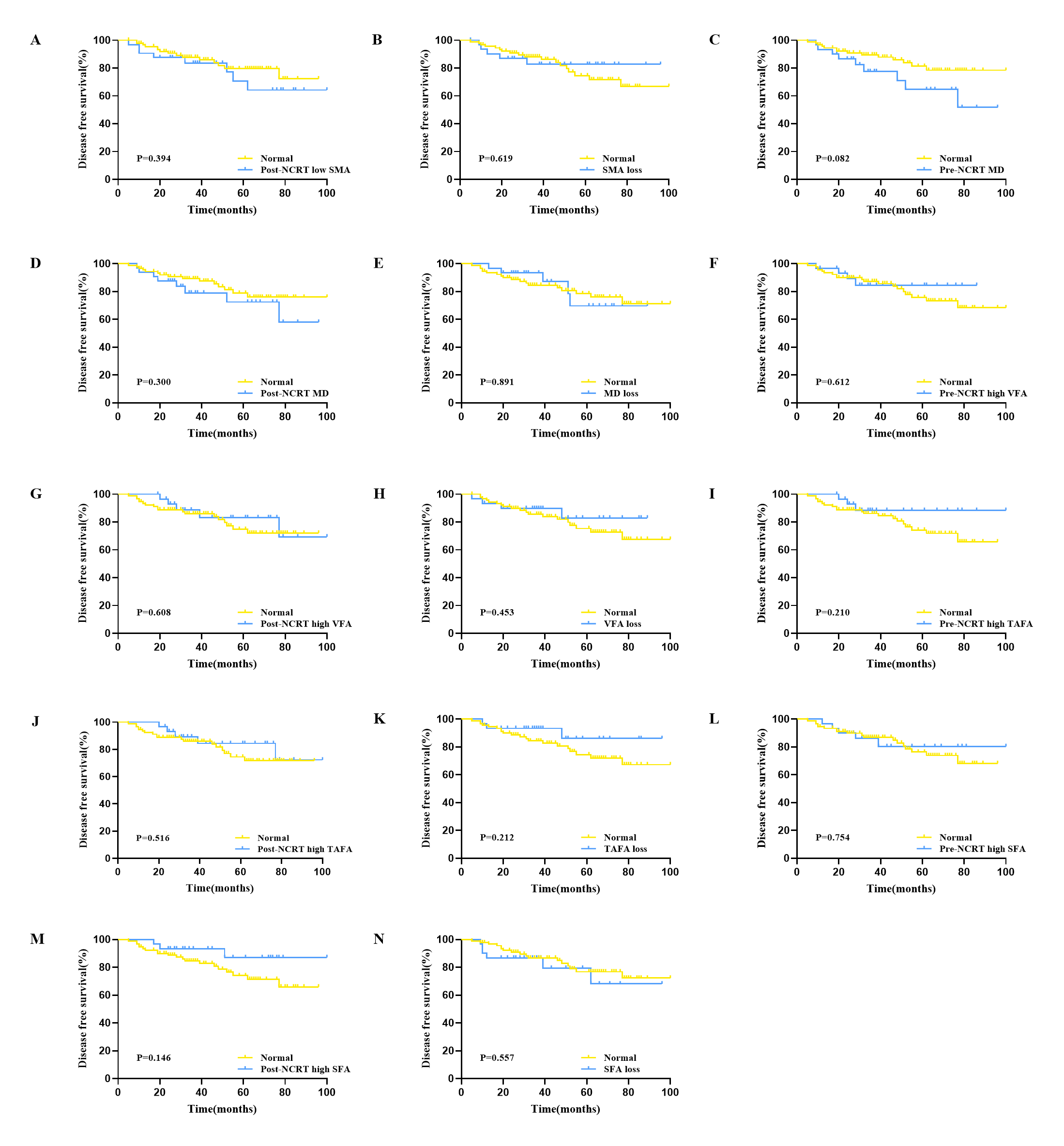

Supplement: Supplementary Figure 3 — Kaplan-Meier analysis for DFS according to body composition and change of body composition. [file Image_3.TIF]

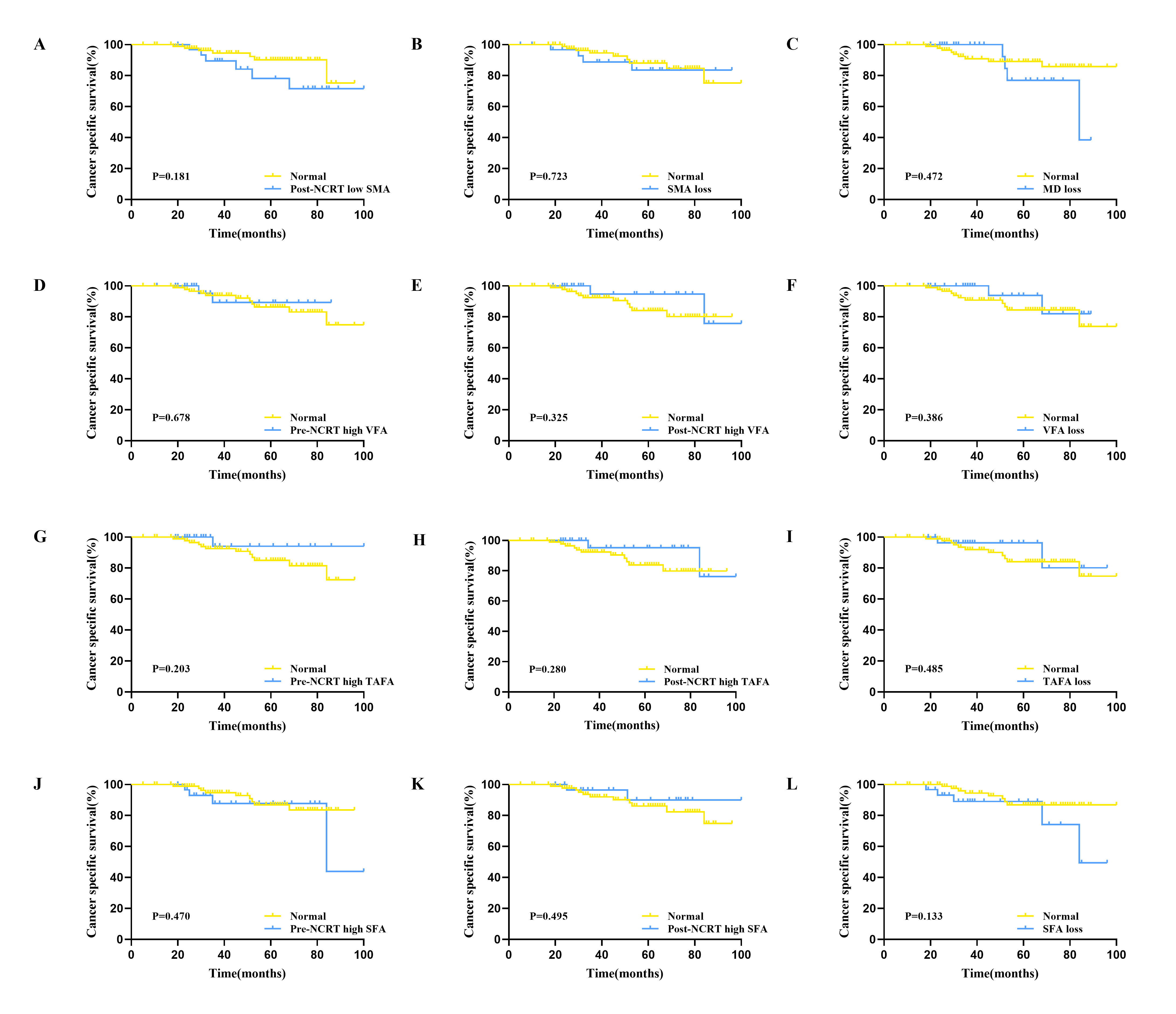

Supplement: Supplementary Figure 4 — Kaplan-Meier analysis for CSS according to body composition and change of body composition. [file Image_4.tif]
