# Supplementary material for: Impact of Body Composition During Neoadjuvant Chemoradiotherapy on Complications, Survival and Tumor Response in Patients With Locally Advanced Rectal Cancer
Source: Front Nutr. 2022 Jan 27;9:796601. doi: 10.3389/fnut.2022.796601 (PMC8830534; doi:10.3389/fnut.2022.796601)
Supplement: Supplementary file 5 [file Table_1.DOCX]

**Supplementary table 1** Patient characteristics

| **Variables** | | | **Total Number (%)** |
| --- | --- | --- | --- |
| **Gender** |  | | |
| Male | | 88 (72.1) | |
| Female | | 34 (27.9) | |
| **Age, years**  **[mean ± SD]** | | 60 ± 12 | |
| **BMI, (kg/m^2^)**  **[mean ± SD]** | | 24 ± 3.2 | |
| **Tumor size** | |  | |
| >4cm | | 63 (45.7) | |
| ≤4cm | | 75 (54.3) | |
| **Tumor location** | |  | |
| Low | | 39 (32.0) | |
| Mid-High | | 83 (68.0) | |
| **Surgery procedures** | |  | |
| Miles | | 41 (33.6) | |
| Dixon | | 67 (54.9) | |
| Hartmann | | 14 (11.5) | |
| **Clinical T stage** | |  | |
| 2-3 | | 98 (80.3) | |
| 4 | | | 24 (19.7) |
| **Clinical N status** | |  | |
| positive | | 91 (74.6) | |
| negative | | 31 (25.4) | |
| **ypTNM** | |  | |
| 0 | | 18 (14.8) | |
| I-III | | 104 (85.2) | |
| **CEA, (ng/L)** | |  | |
| >5 | | 105 (86.1) | |
| ≤5 | | 17 (13.9) | |
| **TRG** | |  | |
| 0-1 | | 72 (59.0) | |
| 2-3 | | 50 (41.0) | |
| **Downstaging** | | 98 (76.6) | |

SD: standard deviation, BMI: body mass index, CEA: carcinoembryonic antigen, TRG: tumor regression grade.
