# Supplementary material for: Impact of Body Composition During Neoadjuvant Chemoradiotherapy on Complications, Survival and Tumor Response in Patients With Locally Advanced Rectal Cancer
Source: Front Nutr. 2022 Jan 27;9:796601. doi: 10.3389/fnut.2022.796601 (PMC8830534; doi:10.3389/fnut.2022.796601)
Supplement: Supplementary file 6 [file Table_2.DOCX]

**Supplementary table 2** Baseline value of body composition

Pre-NCRT: pre-neoadjuvant chemoradiotherapy, Post-NCRT: post- neoadjuvant chemoradiotherapy, IQR: interquartile range, SMA: skeletal muscle area, Hu: Hounsfield unit, VFA: visceral fat area, TAFA: total abdominal fat area, SFA: subcutaneous fat area.

| **Variable** | **Pre-NCRT (IQR)** | **Post-NCRT (IQR)** | **Change of body composition (IQR)** | ***P* value** |
| --- | --- | --- | --- | --- |
| SMA (cm^2^/m^2^) | 46.47(40.05-52.00) | 45.88(39.26-50.69) | -2.01(-5.77-2.58) | 0.17 |
| MD (HU) | 37.04(30.85-41.57) | 37.75(31.96-42.18) | 2.73(-4.35-9.61) | 0.07 |
| VFA (cm^2^/m^2^) | 48.99(28.78-71.20) | 46.93(30.77-70.95) | 1.51(-11.95-17.45) | 0.25 |
| TAFA (cm^2^/m^2^) | 103.12(31.24-61.89) | 104.20(63.52-131.15) | 1.99(-10.18-17.45) | 0.16 |
| SFA (cm^2^/m^2^) | 43.46(31.24-61.89) | 45.35(31.45-61.76) | 1.07(-10.41-18.68) | 0.21 |
