# Supplementary material for: Impact of Body Composition During Neoadjuvant Chemoradiotherapy on Complications, Survival and Tumor Response in Patients With Locally Advanced Rectal Cancer
Source: Front Nutr. 2022 Jan 27;9:796601. doi: 10.3389/fnut.2022.796601 (PMC8830534; doi:10.3389/fnut.2022.796601)
Supplement: Supplementary file 7 [file Table_3.DOCX]

| **Variable** | **BMI** | **ALB** | **FIB** | **HB** |
| --- | --- | --- | --- | --- |
|  | ***P* value** | ***P* value** | ***P* value** | ***P* value** |
| **Pre-NCRT** |  |  |  |  |
| Low SMA | **<0.001** | 0.167 | **0.041** | **0.005** |
| Low MD | 0.632 | 0.475 | 0.918 | 0.462 |
| High VFA | **<0.001** | 0.157 | 0.486 | **0.009** |
| High TAFA | **<0.001** | 0.848 | 0.823 | 0.291 |
| High SFA | **<0.001** | 0.826 | 0.657 | 0.091 |
| **Post-NCRT** |  |  |  |  |
| Low SMA | **<0.001** | 0.082 | 0.423 | **0.006** |
| Low MD | 0.757 | 0.364 | 0.455 | 0.165 |
| High VFA | **<0.001** | 0.785 | 0.619 | 0.303 |
| High TAFA | **<0.001** | 0.396 | 0.845 | 0.413 |
| High SFA | **<0.001** | 0.588 | 0.604 | 0.136 |
| **Change** |  |  |  |  |
| SMA loss | 0.632 | 0.430 | 0.273 | 0.067 |
| MD loss | 0.830 | 0.130 | 0.871 | 0.408 |
| VFA loss | 0.061 | 0.387 | 0.615 | 0.982 |
| TAFA loss | 0.243 | 0.880 | 0.811 | 0.930 |
| SFA loss | 0.162 | 0.359 | 0.283 | **0.025** |

**Supplementary Table 3** Correlation of body composition and nutritional status

BMI: body mass index, ALB: albumin, FIB: fibrinogen, HB: hemoglobin, SMA: skeletal muscle area, MD: muscle density, VFA: visceral fat area, TAFA: total abdominal fat area, SFA: subcutaneous fat area.
